# Histone Deacetylase Inhibition Decreases Cholesterol Levels in Neuronal Cells by Modulating Key Genes in Cholesterol Synthesis, Uptake and Efflux

**DOI:** 10.1371/journal.pone.0053394

**Published:** 2013-01-10

**Authors:** Maria João Nunes, Miguel Moutinho, Maria João Gama, Cecília M. P. Rodrigues, Elsa Rodrigues

**Affiliations:** 1 Research Institute for Medicines and Pharmaceutical Sciences (iMed.UL), Faculty of Pharmacy, University of Lisbon, Lisbon, Portugal; 2 Department of Biochemistry and Human Biology, Faculty of Pharmacy, University of Lisbon, Lisbon, Portugal; University of Pecs Medical School, Hungary

## Abstract

Cholesterol is an essential component of the central nervous system and increasing evidence suggests an association between brain cholesterol metabolism dysfunction and the onset of neurodegenerative disorders. Interestingly, histone deacetylase inhibitors (HDACi) such as trichostatin A (TSA) are emerging as promising therapeutic approaches in neurodegenerative diseases, but their effect on brain cholesterol metabolism is poorly understood. We have previously demonstrated that HDACi up-regulate *CYP46A1* gene transcription, a key enzyme in neuronal cholesterol homeostasis. In this study, TSA was shown to modulate the transcription of other genes involved in cholesterol metabolism in human neuroblastoma cells, namely by up-regulating genes that control cholesterol efflux and down-regulating genes involved in cholesterol synthesis and uptake, thus leading to an overall decrease in total cholesterol content. Furthermore, co-treatment with the amphipathic drug U18666A that can mimic the intracellular cholesterol accumulation observed in cells of Niemman-Pick type C patients, revealed that TSA can ameliorate the phenotype induced by pathological cholesterol accumulation, by restoring the expression of key genes involved in cholesterol synthesis, uptake and efflux and promoting lysosomal cholesterol redistribution. These results clarify the role of TSA in the modulation of neuronal cholesterol metabolism at the transcriptional level, and emphasize the idea of HDAC inhibition as a promising therapeutic tool in neurodegenerative disorders with impaired cholesterol metabolism.

## Introduction

Brain cholesterol is an essential component of neuronal cell membranes and myelin sheets and is involved in several neuronal cellular functions, such as synaptogenesis and synaptic plasticity [1]. Therefore, it is not surprising that increasing evidence relates dysfunction in cholesterol metabolism to the aetiology of many neurodegenerative disorders. For instance, the major risk factor for Alzheimer's disease (AD) is the presence of the E4 isoform of apolipoprotein E, the major cholesterol transporter in the brain [2], while in Niemann-Pick type C (NPC) disease, mutations in the NPC1 and 2 proteins that affect intracellular cholesterol trafficking, are responsible for the pathology [3]. Moreover, cholesterol levels influence amyloid precursor protein processing; high cholesterol levels shift amyloid precursor protein processing towards production of the amyloid-β peptide, which in turn accumulates in neuritic plaques in AD [4].

Due to the blood brain barrier, cholesterol metabolism in the central nervous system (CNS) is distinct from that in other tissues. In fact, the brain is unable to take-up cholesterol from circulation and relies completely on *de novo* synthesis [5]. In the developing CNS, cholesterol synthesis is relatively high, but declines to low levels in the adult [6], mainly due to a highly efficient recycling of brain cholesterol. Despite the efficiency of the cholesterol recycling machinery, the rate of cholesterol synthesis in the adult brain is larger than the rate of accumulation. Therefore, the brain relies on the conversion of cholesterol into 24(S)-hydroxycholesterol (24OHC) as the major mechanism of cholesterol elimination [6], [7], [8]. The enzyme responsible for 24(S)-hydroxylation of cholesterol is a cytochrome P450, CYP46A1, almost exclusively expressed in neurons [9]. Interestingly, inactivation of Cyp46a1 was associated with a selective reduction of cholesterol synthesis [10], while a significant increase in several cholesterol precursors was observed in the brain of Cyp46a1 transgenic mice [11]. This suggests a close relation between synthesis and catabolism of cholesterol in the CNS.

The human CYP46A1 5′- flanking region has been characterized [12], [13]. Unlike other P450 genes, CYP46A1 expression is not dependent on its substrate level, and the promoter is unresponsive to a large number of ligands for different nuclear receptors [12]. Nevertheless, we have demonstrated that CYP46A1 is significantly up-regulated during differentiation of human neuronal cells [14], [15], and that chromatin-modifying agents, 5′-Aza-2′-deoxycytidine and trichostatin A (TSA), dramatically increase CYP46A1 transcription [16], [17]. These latter results suggest that histone deacetylase inhibitors (HDACi) can eventually be used to modulate brain cholesterol metabolism.

HDACs play a key role in histone acetylation homeostasis and in the regulation of fundamental cellular activities, such as transcription. A wide range of brain disorders is associated with imbalanced protein acetylation and treatment with HDACi has been shown to correct these deficiencies and has emerged as a promising new strategy for therapeutic intervention in neurodegenerative diseases. Namely, HDACi have been shown to have neuroprotective, neurotrophic and anti-inflammatory properties, while improving neurological performance and learning/memory in several disease animal models of Huntington's disease [18], [19], [20], spinal muscular atrophy [21], [22], amyotrophic lateral sclerosis [23], [24], [25], and experimental autoimmune encephalomyelitis [26]. Nevertheless, there is hardly any information about how pharmacological intervention in this pathway affects brain cholesterol metabolism. Only recently have HDACi been shown to correct cholesterol storage defects in human NPC1 mutant fibroblasts [27].

Herein, we show that treatment of SH-SY5Y neuroblatoma cells with the pan-HDACi TSA decreases cholesterol levels by inducing an increase in the expression of genes involved in cholesterol efflux and catabolism and a decrease in the transcription of cholesterologenic genes. Moreover, by treating cells with the chemical compound U18666A, which can mimic the accumulation of cholesterol in late-endosomal/lysosomal compartments observed in cells from NPC patients [28], we have confirmed that TSA can ameliorate the phenotype induced by pathological cholesterol accumulation, by restoring the expression of key genes in cholesterol synthesis, uptake and efflux.

## Materials and Methods

### Reagents and antibodies

The chemical inhibitors TSA and U18666A {3-β-[2-(diethylamino)ethoxy]androst-5-en-17-one} were from Sigma (Sigma Aldrich Inc., St Louis, MO, USA). Filipin III (Sigma-Aldrich) and the primary antibodies anti-βIII-tubulin (Covance), anti-lysosome-associated membrane protein 2 (LAMP-2) and anti-glyceraldehyde 3-phosphate dehydrogenase (GAPDH) (Santa Cruz Biotechnology Inc., Santa Cruz, CA, USA) and anti-acetyl-histone 4 (AcH4) (06-598, Millipore, Bedford, MA, USA) were used. The secondary antibodies anti-mouse Alexa Fluor 568 and anti-rabbit Alexa Fluor 488 (Molecular Probes®, Invitrogen, Carlsbad, CA, USA) were used in the immunostaining.

### Cell culture

The SH-SY5Y human neuroblastoma cell line was maintained as previously described [13]. Briefly, cells were maintained in low glucose DMEM (Sigma-Aldrich), at 37°C in humidified 5% CO_2_. The media was supplemented with 10% heat inactivated fetal bovine serum, 2 mM L-glutamine, 100 units/mL penicillin and 100 µg/mL streptomycin (Gibco®, Invitrogen). NTERA-2cl.D1 (NT2) testicular embryonal carcinoma cells were cultured and differentiated as formerly described [14], [15].

### Expression analysis

Total cell RNA was extracted using Trizol Reagent (Invitrogen) following manufacturer's instructions. Real-Time PCR (qPCR) analysis for 3-hydroxy-3-methylglutaryl-coenzyme A reductase (HMGCR) and synthase (HMGCS), mevalonate kinase (MVK), sterol regulatory element-binding protein (SREBP) 1c and 2, low-density lipoprotein receptor (LDLR), apolipoprotein E (APOE), Niemann-Pick disease, type C1 (NPC1) protein and ATP-binding cassette transporter A1 (ABCA1) was performed using SYBR green Master Mix in an ABI 7300 sequence detection system (Applied Biosystems, Foster City, CA, USA) and specific primers (Table 1). CYP46A1 mRNA levels were determined as previously described [17]. Results presented were obtained from at least three individual experiments and each sample was assayed in triplicate. The mRNA levels were normalized to the level of β-actin and expressed as fold change from controls, using the ΔΔCt method. Statistical analysis was performed using ΔCt values.

**Table 1:** List of primers used for qPCR.

Gene	Sequence (5′ – 3′)/source
CYP46A1	CYP46A1 HS00198510_M1 Ref: 4331182
	(Taqman®, Applied Biosystems)
β – actin	ACTB HS99999_M1 Ref.: 4310881E-060521
	(Taqman®, Applied Biosystems)
HMGCR	5′ ATAGGAGGCTACAACGCCCAT 3′ (fwd)
	5′ TTCTGTGCTGCATCCTGTCC 3′ (rev)
HMGCS	5′ GGCACAGCTGCTGTCTTCAAT 3′ (fwd)
	5′ ACCAGGGCATACCGTCCAT 3′ (rev)
MVK	5′ CTCCGATACCATCAAGGG 3′ (fwd)
	5′ GCTCACACTCCAGGGAGA 3′ (rev)
LDLR	5′ CAGATATCATCAACGAAGC 3′ (fwd)
	5′ CCTCTCACACCAGTTCACTCC 3′ (rev)
SREBP1c	5′ GGAGGGGTAGGGCCAACGGCCT 3′ (fwd)
	5′ CATGTCTTCGAAAGTGCAATCC 3′ (rev)
SREBP2	5′ CAGCTGCACATCACAGGGAA 3′ (fwd)
	5′ GTACATCGGAACAGGCGGAT 3′ (rev)
APOE	5′ GACTGGCCAATCACAGGC 3′ (fwd)
	5′ CTGTCTCCACCGCTTGCTC 3′ (rev)
NPC1	5′ ACACCTTCTCTCTCTTTGCGGG 3′ (fwd)
	5′ GCTTGTTCCATCTTCAGCACCTC 3′ (rev)
ABCA1	5′ CCTGTTTCCGTTACCCGACTC 3′ (fwd)
	5′ ACAGGCGAGCCACAATGG 3′ (rev)
β – actin	5′ CTGGAACGGTGAAGGTGACA 3′ (fwd)
	5′ AAGGGACTTCCTGTAACAATCCA 3′ (rev)

### Western blot analysis

Cells were harvested and resuspended in lysis buffer (50 mM Tris-HCl, pH 8.0, 150 mM NaCl, 1% Triton-×100) containing 1 mM DTT and a protease inhibitor mixture (Roche Diagnostics GmbH, Penzberg, Germany). After incubation at 4°C, for 30 minutes, samples were sonicated four times for 4 seconds each, on ice, followed by centrifugation at 12000 *g* for 15 min at 4°C. Proteins were subject to SDS-PAGE gels, electroblotted onto Immobilon P (IPVH00010, Millipore) and incubated with specific antibodies. Results were quantified using the Quantity One version densitometry analysis software (Bio-Rad Laboratories Inc., Hercules, CA, USA).

### Immunocytochemistry

Immunocytochemistry experiments were performed as described previously [29]. Cellular cholesterol was stained using 25 µg/ml filipin III in phosphate-buffered saline for 2 hours at room temperature. In these experiments the AcH4 antibody was used for nuclear staining. Control experiments for non-specific binding were performed in parallel by omission of the primary antibody. Fluorescence visualization was performed in an AxioScope.A1 microscope (Zeiss, Germany) with an AxioCam HRm camera (Zeiss). Fluorescence and co-localization quantification was performed with ImageJ 1.46 software.

### Total cholesterol levels

Total cell cholesterol was quantified using Amplex® Red cholesterol assay kit (Invitrogen) according to the manufacture's instructions. Briefly, cells were resuspended in 1× reaction buffer, placed on ice for 30 min and sonicated. Samples were then diluted in 1× reaction buffer, and 50 µl were used to quantify cholesterol. Samples were placed in a 96-well plate and the reaction was initiated by adding 50 µl of the Amplex® Red reagent/HRP/cholesterol oxidase/cholesterol esterase working solution to each well. The reactions were incubated for 30 min at 37°C. Fluorescence measurements were performed in a spectrofluorometer Infinite® M200 (Tecan, Männedorf, Switzerland). Results presented were obtained from at least three individual experiments and each sample was assayed in triplicate. Cholesterol levels were normalized with total protein levels and expressed as ng of cholesterol per µg of protein.

## Results

### Expression pattern of genes involved in cholesterol synthesis, uptake and efflux after TSA treatment of SH-SY5Y neuronal cells

We have previously shown that inhibition of HDAC activity by sodium butyrate, valproic acid and TSA can significantly up-regulate CYP46A1 gene expression [17], and therefore could eventually be used to modulate brain cholesterol metabolism. Thus, the goal of this study was to evaluate the effect of HDAC inhibition in the expression levels of other key genes in cholesterol metabolism, namely those involved in synthesis, uptake and efflux.

**Figure 1:**
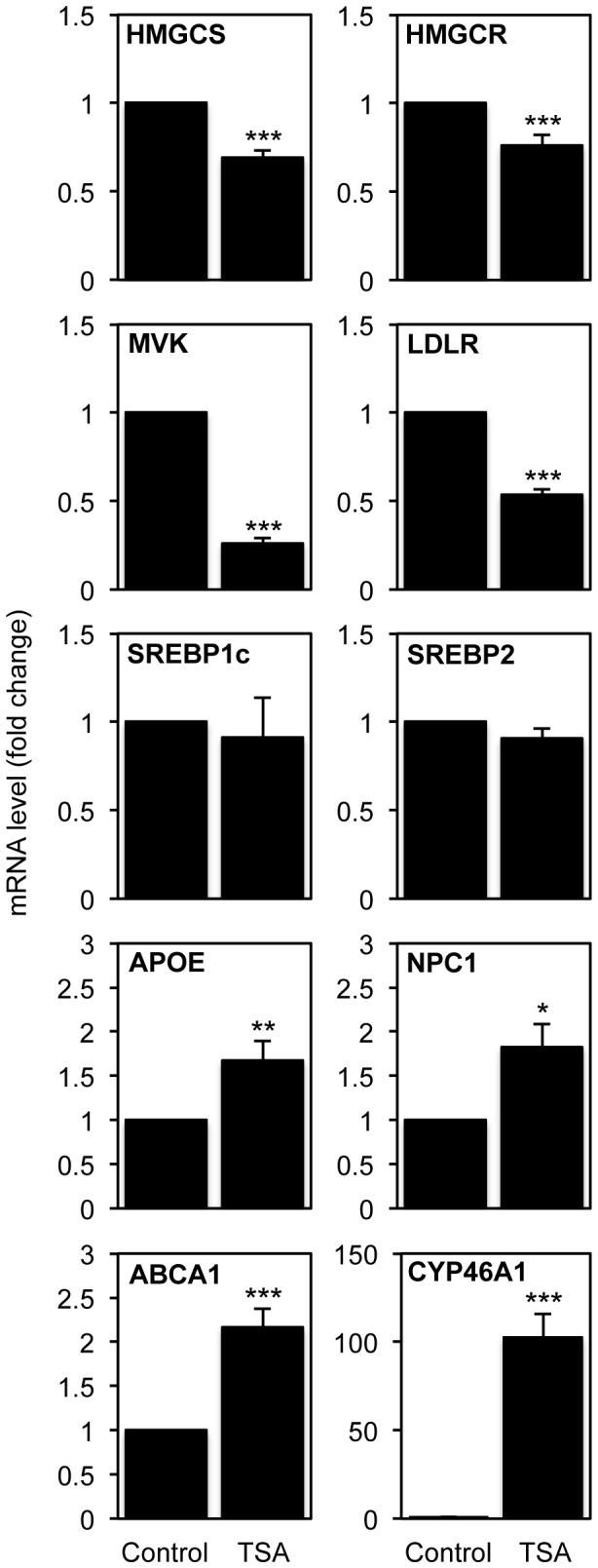
mRNA expression levels of genes involved in cholesterol metabolism after TSA treatment. SH-SY5Y neuroblastoma cells were treated with 250 nM TSA for 16 h. mRNA transcript levels of HMGCS, HMGCR, MVK, LDLR, SREBP1c and 2, APOE, NPC1, ABCA1 and CYP46A1 were analyzed by real-time RT-PCR. Values were normalized to the internal standard β-actin and expressed as fold change relative to untreated cells. Values represent means ± SEM from at least three individual experiments (* *p*<0.05, ** *p*<0.01, *** *p*<0.001).

SH-SY5Y cells were cultured in the absence or presence of 250 nM TSA for 16 h. Total RNA was extracted and mRNA levels were quantified by qPCR. Our results show a significant modulation in the expression levels of genes coding for proteins of the mevalonate pathway, namely HMGCS, HMGCR and MVK, which were decreased to about 70%, 75% and 30% of control values, respectively (Fig. 1). Moreover, TSA also induced a significant 50% down-regulation of the LDLR gene. Since cholesterol homeostasis is transcriptionally controlled by members of the SREBP family of transcription factors [30], we also determined the expression of SREBP1c and SREBP2. Nevertheless, we did not detect any significant changes in the SREBPs mRNA levels. Interestingly, while TSA seems to decrease biosynthesis and uptake, it has the opposite effect on cholesterol efflux, since it induces a significant 2-fold increase in the mRNA levels of the ABCA1 gene. Moreover, a significant increase was also observed in the expression levels of the APOE (1.7-fold) and NPC1 (1.9-fold) genes, coding for proteins responsible for intercellular and intracellular cholesterol transport, respectively. In parallel, we have confirmed our previous results showing a significant increase in CYP46A1 mRNA after TSA treatment (Fig. 1).

**Figure 2:**
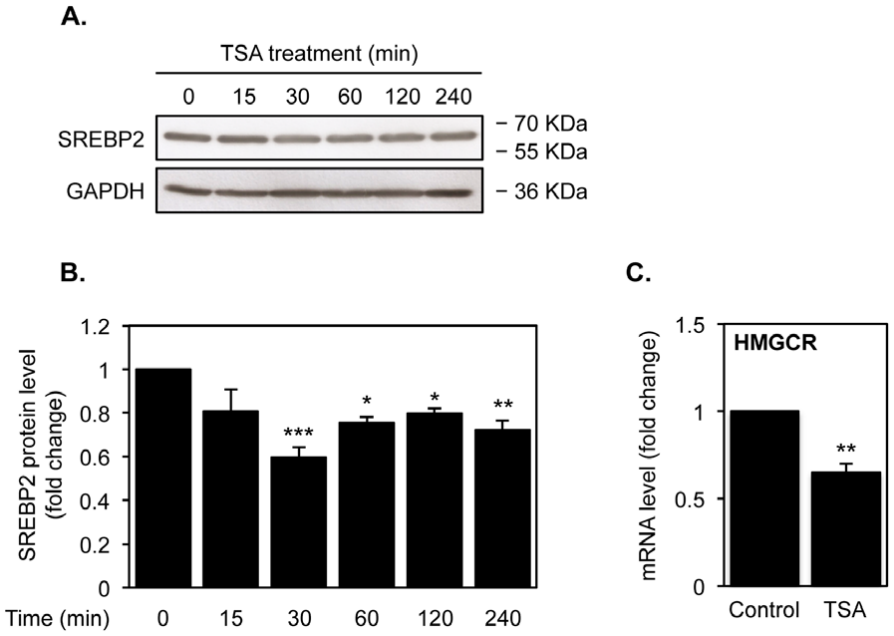
HDAC inhibition by TSA decreases SREBP2 proteolytic cleavage. A) Western blot analysis of SREBP2 protein levels in SH-SY5Y cells after treatment with 250 nM TSA for the indicated time points. The levels of GAPDH are shown as a loading control. B) Quantification of the relative levels of SREBP2 after TSA treatment. Results are expressed as fold change relative to vehicle-treated cells and represent means ± SEM from at least three individual experiments. C) NT2-N *post-mitotic* neurons were treated with 250 nM TSA or vehicle for 16 h. mRNA transcript levels of HMGCR were analyzed by real-time RT-PCR. Values were normalized to the internal standard β-actin and expressed as fold change relative to vehicle-treated cells. Values represent means ± SEM from at least three individual experiments (* *p*<0.05, ** *p*<0.01, *** *p*<0.001).

Although TSA did not affect SREBPs expression, the fact that we observed a significant decrease in the mRNA levels of different genes that are targets of SREBP2, led us to evaluate the effect of TSA on the protein levels of SREBP2 transcriptionally active form. Indeed, SREBPs are synthesized as endoplasmic reticulum precursors, and are subsequently subjected to proteolytic cleavage into their transcriptionally active form [31]. Western blot analysis of total protein extracts from SH-SY5Y cells treated with 250 nM TSA for different time points revealed that TSA decreases the cleaved form of SREBP2 from 30 min after treatment and onwards (ANOVA F = 6.872, df = 5, *p*<0.001; Dunnett: 30 min *p*<0.001, 60 min *p*<0.05, 120 min *p*<0.05, 240 min *p*<0.01) (Fig. 2A and 2B).

Moreover, due to the fact that we have previously shown that differentiated NT2-N neurons are a suitable model to study human neuronal cholesterol metabolism, and to corroborate the results obtained in the neuroblastoma cell line, we have used these cells to assess if TSA could also affect the expression levels of HMGCR, the cholesterol synthesis rate-limiting enzyme, in human NT2-N *post-mitotic* neurons. Real-time PCR analysis confirmed that treatment with 250 nM TSA for 16 h is also able to inhibit the expression of HMGCR gene to approximately 65% of control transcripts level in NT2-N cells (Fig. 2C).

These results suggest that TSA can modulate cholesterol homeostasis in neuronal cells, since it up-regulates genes involved in cholesterol catabolism (CYP46A1) and efflux (ABCA1), and down-regulates genes involved in cholesterol synthesis (HMGCS, HMGCR and MVK) and uptake (LDLR).

### TSA treatment decreases intracellular total cholesterol levels in SH-SY5Y neuronal cells

**Figure 3:**
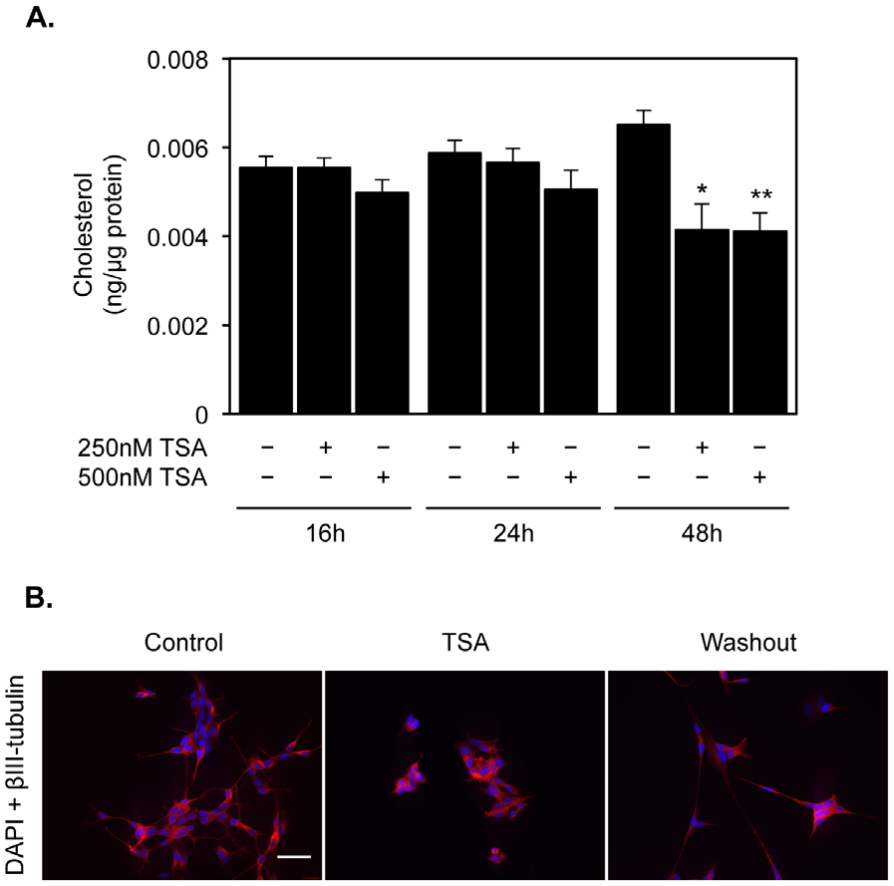
TSA treatment significantly decreases total cholesterol content in neuroblastoma cells. A) Quantification of total cholesterol was performed in SH-SY5Y neuroblastoma cells treated with vehicle, 250 nM TSA or 500 nM TSA for indicated time-points. Values were normalized to total protein content and expressed as ng of cholesterol per µg of total protein. Data represent means ± SEM from at least three individual experiments (* *p*<0.05, ** *p*<0.01). B) βIII-tubulin immunostaining of SH-SY5Y cells treated with vehicle or 250 nM TSA for 48 h, or with 250 nM TSA for 48 h and additionally 48 h without TSA (washout). The results shown are representative of those obtained in at least three independent experiments (*scale bar* = 40 µm).

To determine if the observed changes in the expression levels of genes responsible for cholesterol homeostasis affect total cholesterol content, SH-SY5Y cells were cultured in the absence or presence of 250 nM or 500 nM TSA for 16, 24 and 48 h, and total cholesterol levels were determined with the Amplex® Red cholesterol assay kit in whole-cell fractions (Fig. 3A). Our results showed a significant decrease of 40% in total cholesterol levels 48 h after TSA treatment (Kruskal-Wallis test, F = 24.71, df = 8, *p*<0.01; Dunn: 250 nM TSA 48 h *p*<0.05, 500 nM TSA 48 h *p*<0.01), which appear to be dose-independent.

The TSA-mediated reduction of total cholesterol levels can in part be due to cell toxicity, since viability tests confirmed previously published data [32]. Indeed, our results showed significant lactate dehydrogenase release and a decrease in the ability of cells to convert 3-(4,5-dimethylthiazol-2-yl)-5-(3-carboxymethoxy- phenyl)-2-(4-sulfophenyl)-2H-tetrazolium salt (MTS) into formazan 48 h after TSA treatment (data not shown). Nevertheless, since our analysis did not include cells that were detached from the cell cultures dishes, we decided to evaluate if the TSA effect is reversible upon washout of the drug. To confirm this, we labeled SH-SY5Y cells with the neuronal cytoskeletal marker type III β-tubulin in control and 250 nM TSA-treated cells for 48 h, and in cells submitted to a 48 h washout of the HDACi. Our results show that, as previously reported, SH-SY5Y display small cell bodies and long branched neurites. Treatment with TSA for 48 h induced a retraction of the neurites that was abolished by the washout of TSA, suggesting that the TSA effect is reversible (Fig. 3B).

**Figure 4:**
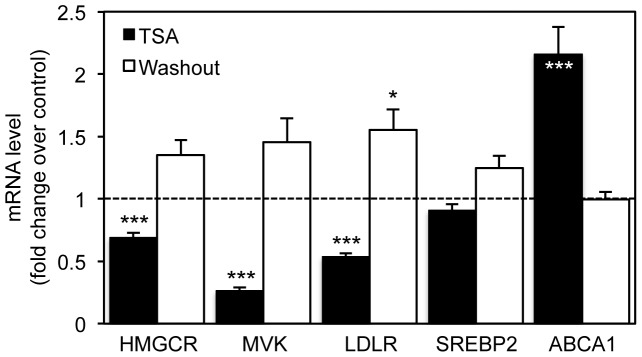
TSA removal completely reverts the transcriptional changes observed after HDAC inhibition. SH-SY5Y neuroblastoma cells were treated with vehicle or 250 nM TSA for 16 h, or with 250 nM TSA for 16 h and additionally 24 h without TSA (washout). mRNA transcript levels of HMGCR, MVK, LDLR, SREBP2 and ABCA1 were analyzed by real-time RT-PCR. Values were normalized to the internal standard β-actin and expressed as fold change relative to vehicle-treated cells. Values represent means ± SEM from at least three individual experiments (* *p*<0.05, *** *p*<0.001).

To further confirm that the TSA effect is reversible we determined the expression levels of HMGCR, MVK, LDLR, SREBP2 and ABCA1 genes in SH-SY5Y cells submitted to a 24 h washout of the drug after treatment with 250 nM TSA for 16 h (Fig. 4). Real-time PCR demonstrated that after TSA withdrawal the expression levels of the analyzed genes return to control levels, clearly suggesting the reversible effect of TSA treatment.

**Figure 5:**
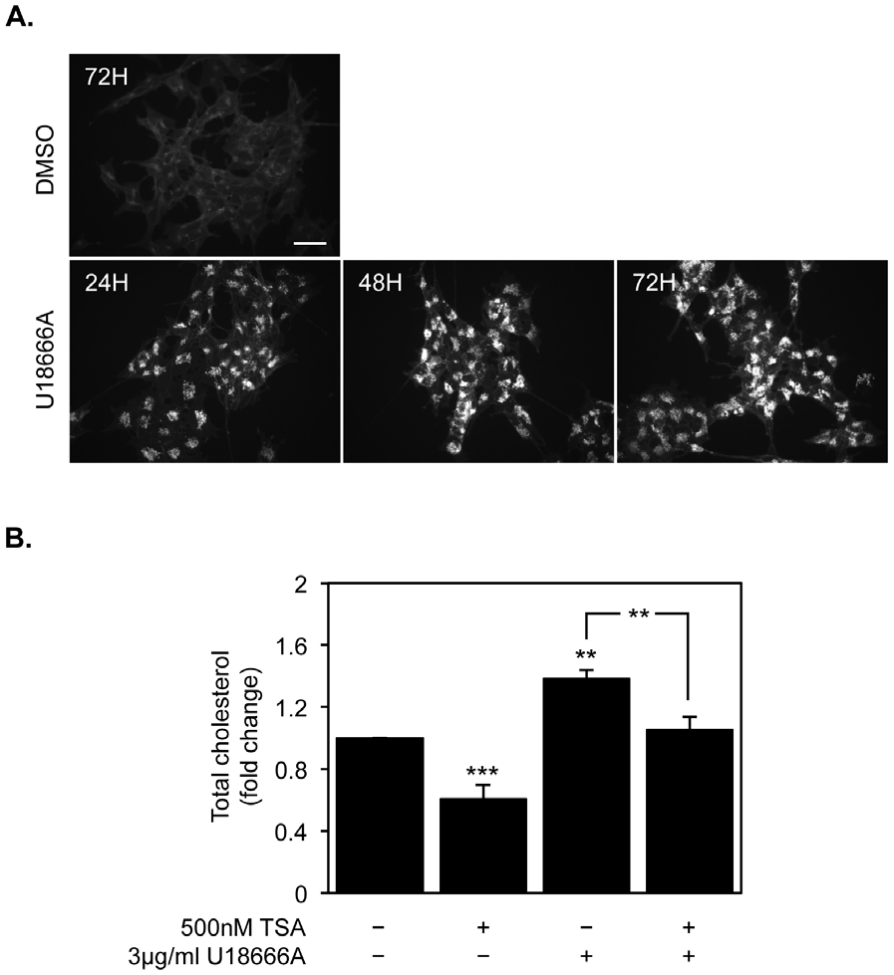
TSA reverts the increase in total cholesterol levels observed after U18666A treatment. A) Filipin III immunofluorescence staining of SH-SY5Y cells treated for the indicated time-points with 1 µg/ml U18666A or vehicle (*scale bar* = 40 µm). B) SH-SY5Y cells were treated with 3 µg/ml U18666A for 24 h and with or without 250 nM TSA for 48 h, and total cholesterol levels were determined. Values were normalized to total protein content and expressed as ng of cholesterol per µg of total protein. Data represent means ± SEM from at least three individual experiments (** *p*<0.01, *** *p*<0.001).

To further confirm that TSA could ameliorate the phenotype induced by pathological cholesterol accumulation, we pre-treated SH-SY5Y neuroblastoma cells with U18666A, a compound that can mimic the accumulation of unesterified cholesterol in late-endosomal/lysosomal compartments similar to that occurring in NPC1-deficient fibroblasts. The dynamics of intracellular cholesterol accumulation was visualized by fluorescence microscopy after filipin staining which can specifically label unesterified cholesterol. Moreover, in parallel we determined changes in total cholesterol levels, as above-mentioned. Untreated cells showed pale and diffuse filipin staining, whereas cells treated with U18666A revealed a characteristic intense and punctuate staining pattern (Fig. 5A). As expected, treatment with 3 µg/ml U18666A induced a significant increase in total cholesterol levels 72 h after treatment (8.66 ng±0.25 of cholesterol/µg of total cell protein vs. 11.86 ng±0.32 of cholesterol/µg of total cell protein) (ANOVA one-way test: *F* = 28.57, df = 3, *p*<0.001; Tukey HSD, *p*<0.01) (Fig. 5B). To confirm that TSA could ameliorate the NPC phenotype, we treated neuroblastoma cells with 3 µg/ml U18666A for 24 h, and then incubated cells for further 48 h in the presence or absence of 500 nM TSA. Interestingly, U18666A-induced cholesterol increase was abolished by TSA treatment (Tukey HSD, *p*<0.01) (Fig. 5B).

### TSA treatment partially reverts the effect U18666A on cholesterol synthesis and efflux and promotes lysosomal cholesterol redistribution

**Figure 6:**
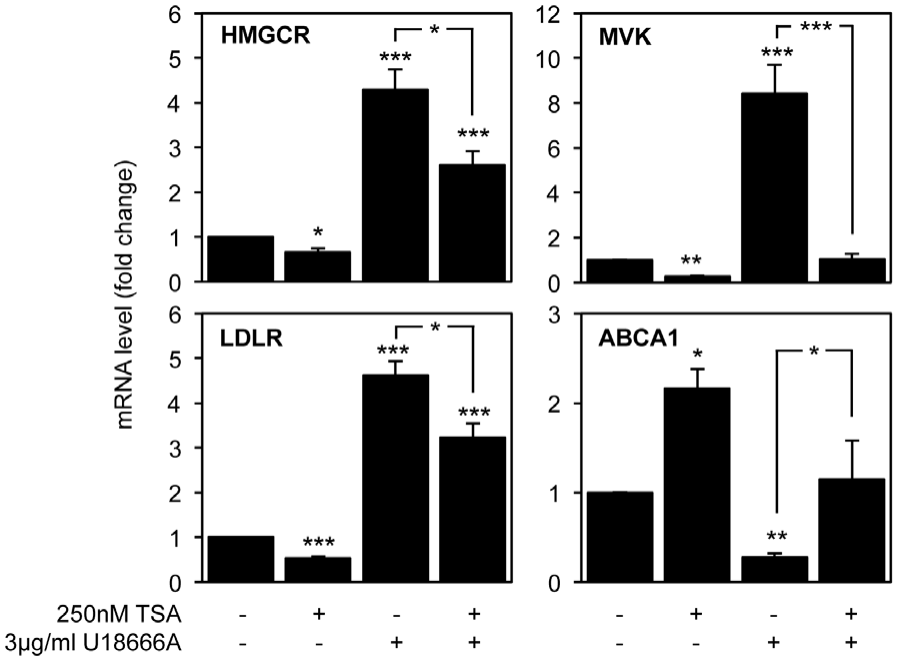
TSA treatment partially reverts the effect of U18666A on the expression of genes involved in cholesterol synthesis, uptake and efflux. SH-SY5Y neuroblastoma cells were pre-treated with 3 µg/ml U18666A for 6 h and with or without 250 nM TSA for 16 h. mRNA transcript levels of HMGCR, MVK, LDLR and ABCA1 were analyzed by qPCR. Values were normalized to the internal standard β-actin and are expressed as fold change relative to untreated cells. Data represent means ± SEM from at least three individual experiments (* *p*<0.05, ** *p*<0.01, *** *p*<0.001).

It has been shown that cholesterol homeostasis in NPC1^−/−^ mice was improved by treatment with valproic acid, a weak HDACi [33]. More recently, HDAC inhibitors led to the correction of the NPC phenotype in cells with either one or two copies of the NPC1^I1061T^ mutation, a correction mainly associated with increased expression of NPC1 protein [27]. This is in accordance with our results that demonstrate the up-regulation of NPC1 gene after TSA treatment. Moreover, since we observed that TSA restores cellular cholesterol content after U18666A treatment, we then evaluated how the expression of genes involved in cholesterol homeostasis was being affected by U18666A treatment, in the presence or absence of TSA. We analyzed the expression levels of six genes, involved in cholesterol synthesis, influx, transport, catabolism and efflux (HMGCR, MVK, LDLR, NPC1, CYP46A1 and ABCA1, respectively), and shown to be influenced by TSA treatment. SH-SY5Y cells were pre-treated with 3 µg/ml U18666A for 6 h, and subsequently with or without 250 nM TSA for 16 h. mRNA levels were detected by qPCR (Fig. 6). Since CYP46A1 and NPC1 mRNA levels were not affect by U18666A treatment (data not shown), we failed to observe if TSA could counteract the cholesterol accumulation induced by U18666A, by modulating the expression of these genes. Nevertheless, our results show that inhibition of intracellular cholesterol traffic induces a significant increase in the expression levels of HMGCR, MVK and LDLR to approximately 4.3-, 8- and 4.5-fold of control levels, respectively (ANOVA F = 76.55, df = 3, *p*<0.001, Tukey HSD *p*<0.001, ANOVA F = 33.799, df = 3, *p*<0.001, Tukey HSD for unequal N *p*<0.001 and ANOVA F = 138.349, df = 3, *p*<0.001, Tukey HSD for unequal N *p*<0.001). Interestingly, TSA can correct the effect of U18666a treatment on MVK expression levels, decreasing mRNA levels to control values. Moreover, TSA can also significantly revert the increase on HMGCR and LDLR mRNA levels induced by U18666a treatment (Tukey HSD *p*<0.05). Moreover, inhibition of intracellular cholesterol traffic significantly down-regulated ABCA1 to 30% of the control values (ANOVA F = 13.599, df = 3, *p*<0.001; Tukey HSD for unequal N *p*<0.01). TSA treatment can also correct this decrease to control levels. Overall, these results clearly demonstrate that TSA partially reverts the effect of intracellular cholesterol accumulation in the expression levels of genes involved in cholesterol homeostasis.

**Figure 7:**
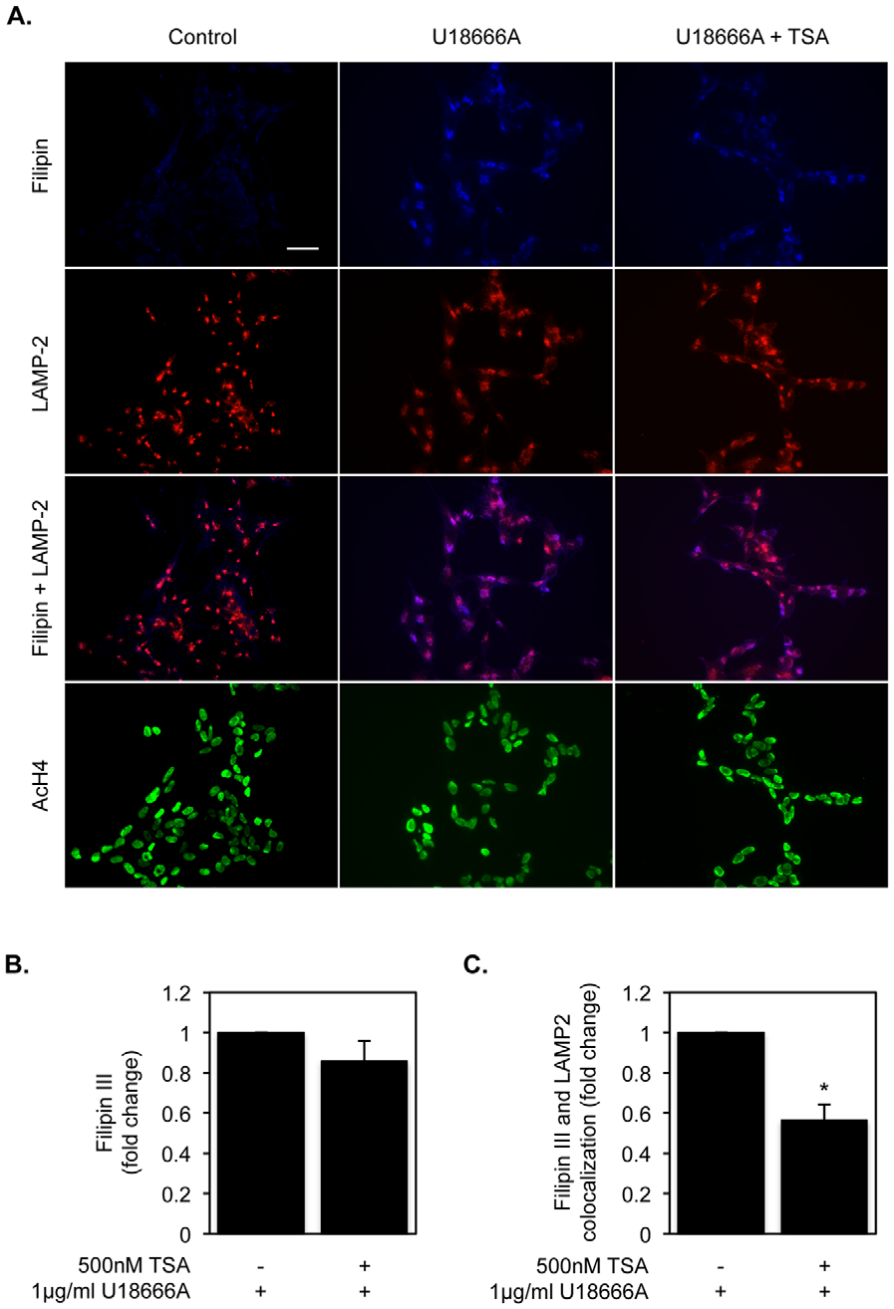
TSA treatment partially reverts the U18666A-induced phenotype by promoting cholesterol redistribution in neuroblastoma cells. A) Filipin III, lysosome-associated membrane protein 2 (LAMP-2) and acetyl-histone 4 (AcH4) immunofluorescence staining of SH-SY5Y cells pre-treated with 1 µg/ml U18666A for 24 h and with or without 250 nM TSA for 16 h. After the 24 h treatment with U18666A, the medium was removed and replaced with new medium without U18666A, and with or without TSA. Colocalization images of filipin III and LAMP-2 immunostaining are also presented. The results shown are representative of those obtained in at least three independent experiments. (*scale bar* = 40 µm). Quantification of filipin III fluorescence (B) and filipin III and LAMP-2 colocalization (C) was performed, the values were normalized to total cell number assessed by AcH4 immunostaining and are expressed as fold change relative to U18666A treated cells. Data represent means ± SEM of at least three independent experiments (* *p*<0.05).

To assess if TSA was able to revert the phenotype of intracellular cholesterol accumulation induced by U18666A in a neuronal derived cell line such as the SH-SY5Y, we performed immunocytochemistry experiments in cells treated with 1 µg/ml U18666A for 24 h, and subsequently with 250 nM TSA or vehicle for 16 h (Fig. 7). Cholesterol accumulation was evaluated by filipin staining and its association with late endosomes/lysosomes was assessed by colocalization with the lysosomal marker LAMP-2. Since our goal was to evaluate the effect of TSA on the improvement of U18666A-induced phenotype, we pre-treated cells with this compound for 24 h, since a significant cholesterol accumulation was already observed at this time point. The culture medium was then removed and new medium with TSA or vehicle was added to cells. Fluorescence quantification after filipin staining indicated that no significant changes in cellular cholesterol loading were detected after 24 h of TSA treatment (Fig. 7B), which is in accordance with the results obtained for total cholesterol levels (Fig. 3A). However, we observed a decrease of approximately 40% in the co-localization of cholesterol and the LAMP-2 marker after TSA treatment (Fig. 7C), which suggests that this HDACi is initially inducing the release of cholesterol from late endosomes/lysosomes and therefore promoting cholesterol redistribution in the cell.

## Discussion

CYP46A1 is a key enzyme in brain cholesterol homeostasis. This neuronal-specific cytochrome P450 mediates the conversion of cholesterol into 24(S)-hydroxycholesterol [9], which represents the major pathway of cholesterol turnover in the CNS [7], [8]. As we have demonstrated in our previous studies that HDACi were able to significantly up-regulate *CYP46A1* transcription levels [17], we hypothesized that these compounds would probably be able to control neuronal cholesterol levels, due to the importance of this enzyme in cholesterol homeostasis. That idea led us to investigate if and how HDAC inhibition was affecting the transcription of other genes involved in cholesterol metabolism. The studies described here clearly demonstrate that, in fact, TSA has the ability to decrease total cholesterol content of neuroblastoma cells by up-regulating genes responsible for cholesterol catabolism (CYP46A1) [17] and efflux (ABCA1), and down-regulating genes responsible for cholesterol synthesis (HMGCR, HMGCS and MVK) and uptake (LDLR). From these genes, *CYP46A1* is the only one that has already been described to respond to HDACi treatment through hyperacetylation of the promoter in neuronal derived cells [17]. HMGCR, HMGCS and MVK were described to be up-regulated by HDACi [34], along with ABCA1 [35], although both studies were performed in non-neuronal cell lines. In accordance with our results on mRNA levels, we have observed that TSA treatment induces a decrease in the proteolytic cleaved transcriptionally active form of SREBP2. This protein is the major transcriptional activator of cholesterol metabolism, which up-regulates the majority of enzymes involved in cholesterol synthesis, including HMGCR, and the uptake of cholesterol by inducing the LDLR expression [31]. Therefore, our results suggest that the TSA effect on cholesterol metabolism is not only due to promoter acetylation/deacetylation-dependent mechanisms.

Since some of the effects of HDAC inhibitors in cancer cells cannot be recapitulated in non-transformed cells, we have corroborated our results, by showing that TSA also reduced the mRNA levels of the HMGCR, the cholesterol synthesis rate-limiting enzyme in human *post-mitotic* NT2-N neurons.

It has recently been reported by two different studies that HDACi treatment can reverse the phenotype of cholesterol accumulation and correct transport defects in human NPC fibroblasts [27], [36]. HDACi effect was suggested to depend on restoration of the expression of the majority of HDACs that were up-regulated in NPC cells [36], or be associated with an increased stabilization of NPC1 protein levels [27]. Nevertheless, the exact mechanisms remain to be determined. In addition, HDACs play important roles in several neurological functions [37], and HDACi have been described to be neuroprotective in various neurodegenerative models [38]. Indeed, overexpression of neuronal specific HDAC2 decreases dendritic spine density, synapse number, synaptic plasticity and memory formation, which are reverted by HDAC inhibition [39]. Interestingly, cholesterol is also essential for synaptogenesis [40], [41] and dendrite outgrowth [42]. Some studies have already identified specific HDACs involved in cholesterol and lipid metabolism. HDAC3 has been described to down-regulate cholesterol synthesis in HeLa cells, through the repression of lanosterol synthase expression, an enzyme of cholesterol biosynthetic pathway [34]. The studies of Munkacsi and coworkers [36] identified a significant up-regulation of HDAC4 in fibroblasts of NPC patients, along with HDAC6 and 11, which was markedly corrected by HDAC inhibition. Several HDAC candidates could mediate the TSA effect; however, their identification can only be achieved by specific chemical inhibition concomitantly with efficient knockdown by siRNA transfection.

Although neurodegeneration is the fatal cause in NPC patients, the majority of experimental approaches are performed in fibroblasts. Interestingly, our results show that TSA reverses the increase in total cholesterol levels induced by treatment of human neuronal cells with U18666A, a chemical compound that leads to the accumulation of cholesterol in late-endosomal/lysosomal compartments observed in cells from NPC patients [28]. Moreover, TSA also reverses the increase in the transcription of genes involved in cholesterol synthesis (HMGCR, MVK) and uptake (LDLR), and a decrease in cholesterol efflux (ABCA1). These changes induced by U18666A are also characteristic of NPC disease. The NPC phenotype of unesterified cholesterol accumulation in late endocytic organelles, as a result of defective cholesterol transport due to mutations in NPC proteins, is also characterized by impaired cholesterol esterification [43] and inefficient suppression of cholesterol synthesis and LDLR activity [44]. The modulation of LDLR is of particular importance since lipoprotein uptake through the LDLR-mediated endocytosis is the major pathway leading to accumulation of unesterified cholesterol in NPC cells [45]. In the context of the CNS, astrocytes cooperate with neurons in the supply of cholesterol in the form of ApoE containing lipoproteins that subsequently are taken up by neurons through the LDLR [46]. Interestingly, restoring the Npc1 in Npc ^−/−^ mice only in astrocytes enhanced survival and decreased neuronal storage of cholesterol and neuronal degeneration [47].

In addition to the end-point decrease in cholesterol content, early time-points of TSA treatment showed a reduction in the amount of free cholesterol associated with late endosomes/lysosomes. This evidence suggests that alterations in the intracellular cholesterol transport induced by HDAC inhibition are facilitating cholesterol removal from the cell. Although we demonstrated that TSA induces NPC1 expression, a protein crucial for intracellular movement of cholesterol out of the late endosomal system and into the cytosol and other organelles, U18666A has no effect on the transcription of this gene, indicating that release of cholesterol from endosomes was not dependent on an increase in NPC1 expression. Nevertheless, improvement of cholesterol movement from late endosomes to the plasma membrane and to the ER could be sensing the cells to cholesterol, leading to a down-regulation of synthesis and uptake, by the SREBP mediated pathway [48]. Therefore, it remains to be determined if TSA directly ameliorates cholesterol accumulation phenotype through transcriptional regulation of specific target genes (HMGCR, MVK, LDLR and ABCA1) or if it indirectly controls cholesterol content by modulating SREBP2 cleavage.

Overall, this study describes how HDACi modulate cholesterol metabolism at the transcriptional level in neuroblastoma cells, thus reinforcing the idea of inhibiting HDACs as an effective therapeutic avenue to target neurodegenerative conditions that lead to cholesterol accumulation.
